# Preparing lifelong learners for delivering pharmaceutical care in an ever-changing world: a study of pharmacy students

**DOI:** 10.1186/s12909-020-02394-w

**Published:** 2020-12-10

**Authors:** Sarah Khamis, Abdikarim Mohamed Abdi, Bilgen Basgut

**Affiliations:** Department of Clinical Pharmacy, Faculty of Pharmacy, Near East University, Nicosia, Northern Cyprus Mersin, 10 Turkey

**Keywords:** Continuing professional development, Lifelong learner, Self-directed learning, Pharmacy education, Competence, global health challenges

## Abstract

**Background:**

Continuing professional development (CPD) continues to gain acceptance as a model for health care professionals to engage in lifelong learning (LLL). Many pharmacy schools have not adopted yet specific programs targeting the development of LLL skills, though LLL is widely accepted as an essential competence. This paper examines the effectiveness and utility of a longitudinal CPD training program.

**Methods:**

A CPD simulation course was introduced to a cohort of fifth year students in Northern Cyprus in the 2018–2019 academic year. The program was delivered as an interactive orientation course in one semester; meanwhile, in the second semester, the students applied the CPD cycle and completed their portfolios during their final experiential practice. A mixed-methods approach was used to evaluate the outcomes of the intervention using students’ preparedness for lifelong learning (SPLLL) self-administered questionnaire delivered pre-post program, focus group sessions for students to reflect on the course experience, and instructors’ evaluations of portfolios.

**Results:**

Following the implementation of the course, students’ assessment scores were significantly higher overall and for all scale domains, including “knowledge, skills, attitude and practice”, compared to the baseline assessment. Additionally, compared to fifth year students who responded to the second SPLLL questionnaire, the intervention group students’ assessment was significantly higher in knowledge, skills, and practice. The qualitative analysis reported high student satisfaction and achievement of the course objectives. Nineteen of the students scored high on their portfolios.

**Conclusion:**

The CPD simulation course provided students with opportunities to practice and develop self-assessment and self-management skills that are all desirable for lifelong learning and prepared them for CPD.

## Background

In an ever changing world, pharmacists among other healthcare professionals are required to continuously embrace new behaviors and adjust their practices toward emerging roles in patient care [1, 2]. Lifelong learning (LLL) and continuing professional development (CPD) remains more than ever critical for both current and future pharmacists, in face of global health challenges, new technologies, services and therapies that are continually and rapidly introduced into their daily practice [2]. For instance, the current global COVID-19 outbreak, with its huge magnitude and severity exposed pharmacists to challenges and practices they never experienced before [3]. Thus, pharmacy undergraduate programs are required to prepare graduate pharmacists with adequate competency to obtain roles in health and wellness promotion [4]. A pharmacist’s high-level specialist knowledge and skills are maintained through an ongoing commitment to LLL [5].

LLL is defined as “all learning activities undertaken throughout life, with the aim of improving knowledge, skills and competences within a personal, civic, and social and/or employment-related perspective” [6]. It assures continuing competence whereby individuals consider learning and practice to be continuous, beginning with first year university studies through advanced practice [7].

The approach or framework for achieving LLL for practitioners in the United States (US) and many other countries is through CPD [8]. CPD is designed to be a self-directed, practitioner-centered, and outcome-based learning process to meet the specific goals and objectives of individual pharmacists, ultimately improving patient and public health outcomes [8]. CPD is an ongoing cyclical process involving the following: self-appraisal, developing a personal learning plan, taking action or implementing a learning plan, and evaluation [8].

LLL and CPD are among the core competencies targeted in modern pharmacy curricula and addressed in the International Pharmaceutical Federation (FIP) global pharmacy education vision and standards released at the end of 2016 [8, 9]. Core competences should be initiated, developed and assessed within curricula to assure that graduates possess them in practice. Thus, these assure that CPD ready graduates and students are not only introduced to CPD principles but also required to practice them within their learning environments [10].

However, introducing these concepts into pharmacy curricula and students’ practice is challenging since implementation strategies differ considerably between institutions [10, 11]. Several studies have evaluated different cocurricular activities and experiences related with CPD among doctor of pharmacy (PharmD) students in the US both preceding and following the release of the Accreditation Council for Pharmacy Education (ACPE) standards of 2016. These studies showed the benefits of electronic portfolios [12], other self-assessment and self-reflection activities [13], live and online CPD training courses [14, 15], educating students on how to write SMART goals [15], and adopting a monthly seminar or a journal club for the acquisition of CPD or LLL skills [9]. Earlier attempts involve Daniel L. et al. (2001) introduction of a self-directed professional development program implemented within internal medicine rotations. The aim was to prompt students to take responsibility for their own professional growth and develop LLL habits [16].

Tofade T. et al.(2011) proposed the integration of CPD throughout curriculum [15]. In the proposed model, students are introduced to CPD through CPD lectures and training in the early years and are then requested to submit a CPD plan and updated portfolio routinely until graduation [15]. This model in line with the Kolb’s Cycle in which an effective learning requires the learner to progress through the cycle of concrete learning, reflective observation, abstract conceptualization and active experimentation [17]. Few studies reported implementing such a longitudinal program in pharmacy schools, namely, the Roseman University College of Pharmacy (RUCOP) longitudinal CPD program for a cohort of PharmD students in the US [18] and the traffic light report (TLR) program implemented within a Bachelor’s of Science (BSc) in pharmacy curricula as an elective course in an Australian university [19].

The RUCOP program involved CPD as part of the didactic curriculum of their three-year PharmD program and the experiential year. As a result, students’ oral, written and interprofessional communication, leadership, and time management skills were reported to be improved over the course [18]. Other schools in the US evaluated implementation of CPD in either first [14] or final [16] didactic years only or within an experiential practice course [15, 19].

The TLR was a two semester program designed to provide students with a form of sustainable assessment drawn on two facets of CPD, specifically, self-assessment and the national competence standards, both of which are essential to a pharmacists’ LLL [19]. The program was reported to provide pharmacy students with an opportunity to practice self-assessment skills, though poor student acceptance of the TLR was reported [19]. An earlier study at the University of Central Lancashire in the UK also reported poor outcomes when introducing a CPD activity similar to that for pharmacists in a master of pharmacy (M.pharm) degree program [20]. Tofade T. et al.(2011) stated that the difference in the CPD implementation outcomes between the US PharmD schools and other countries was due the nature of PharmD programs and students being advanced compared to M.pharm or BSc in pharmacy programs elsewhere; thus, PharmD students may find the CPD process easier to grasp [15].

Donald Kirkpatrick developed a four-level learning evaluation hierarchy that’s commonly used to evaluate the effectiveness of educational programs. This model identifies the following four levels as evidence for learning that is reaction, learning, behavior, and results [21]. Outcomes utilized in the above mentioned CPD programs fall within the first three levels of the Kirkpatrick hierarchy.

The multifaceted nature of CPD as an advanced model compared to traditional approaches to continuing education (CE) necessitate that pharmacists must receive training and guidance in order to develop the required competence and implement the CPD process in their practices [22, 23].

Other countries around the world currently have a variety of systems in place for CE in pharmacy [22], spanning from traditional CE requirements to the full implementation of a more extensive CPD approach [22]. Conversely, the situation was no or poor programs are adopted to develop LLL and CPD associated skills is also present in schools. This may further explain why implementing CPD programs is challenging outside the states [19].

In Turkey and Northern Cyprus, CPD programs are not objectively structured or a compulsory requirement for recertification in pharmacy practice. As a result, pharmacists that are preceptors for new graduates are unfamiliar with the CPD process since most of them were not exposed to it [24, 25].

There are over 40 pharmacy faculties in Turkey and Northern Cyprus, with local accreditations awarded by the Turkish Higher Education Counsel for professional 5 year programs [26]. Out of these, Near East University (NEU) is certified by the ACPE [27]. To acquire this certification, the faculty of pharmacy reviewed its curriculum in order to meet the required standards. CPD and LLL were among the competencies targeted to be achieved by students enrolled in the M.pharm program that the faculty offers.

### Objective

To our knowledge, there are no studies that have evaluated the implementation of a CPD simulation model in developing countries. Many universities worldwide are currently acquiring an ACPE certification, which requires addressing CPD in curricula and educational program outcomes. This study fills this research gap by examining the effectiveness and utility of a longitudinal CPD training program introduced to fifth year M.pharm students in North Cyprus.

The hypothesis of this research is that a CPD simulation program is providing opportunities to practice and develop skills in self-assessment and awareness, SMART planning and monitoring, and documentation of one’s own learning plans and activities, all of which are desirable for LLL.

## Methods

A CPD simulation course was introduced to a cohort of fifth year pharmacy students at NEU in Northern Cyprus through the 2018–2019 academic year. The course objective was to improve students’ competence in CPD and LLL through an interactive orientation course in the first semester followed by a self-directed learning (SDL) assignment required from each student during their final experiential practice.

**Fig. 1:**
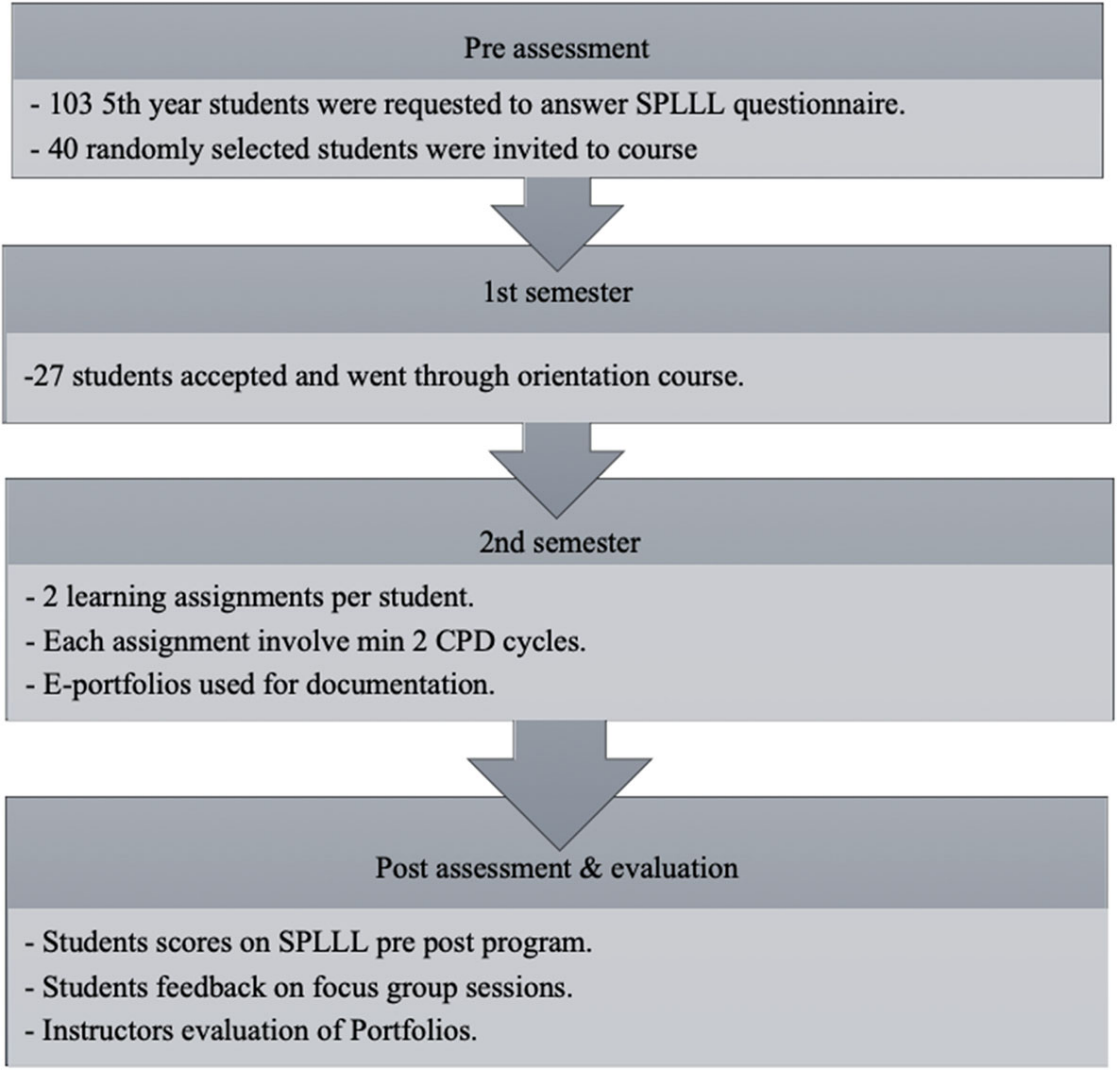
Study Design & Flow

A mixed-method design was adopted to evaluate the implementation outcomes. Students’ preparedness for CPD and LLL was assessed using students’ preparedness for lifelong learning (SPLLL) self-administered questionnaire, which was developed and validated by the research group, and delivered pre-post program. Students’ feedback was also evaluated using an exploratory qualitative approach from a focus group with the students at the end of the study period. Each student was required to reflect on and document his learning using a student portfolio, which was also evaluated by the instructors (see Fig. 1).

### Design and implementation

The course was launched as a longitudinal elective course named the CPD course. The course instructors received prior training in CPD and LLL skills development conducted by experts from the ACPE and a pharmacy education consulting company.

**Fig. 2:**
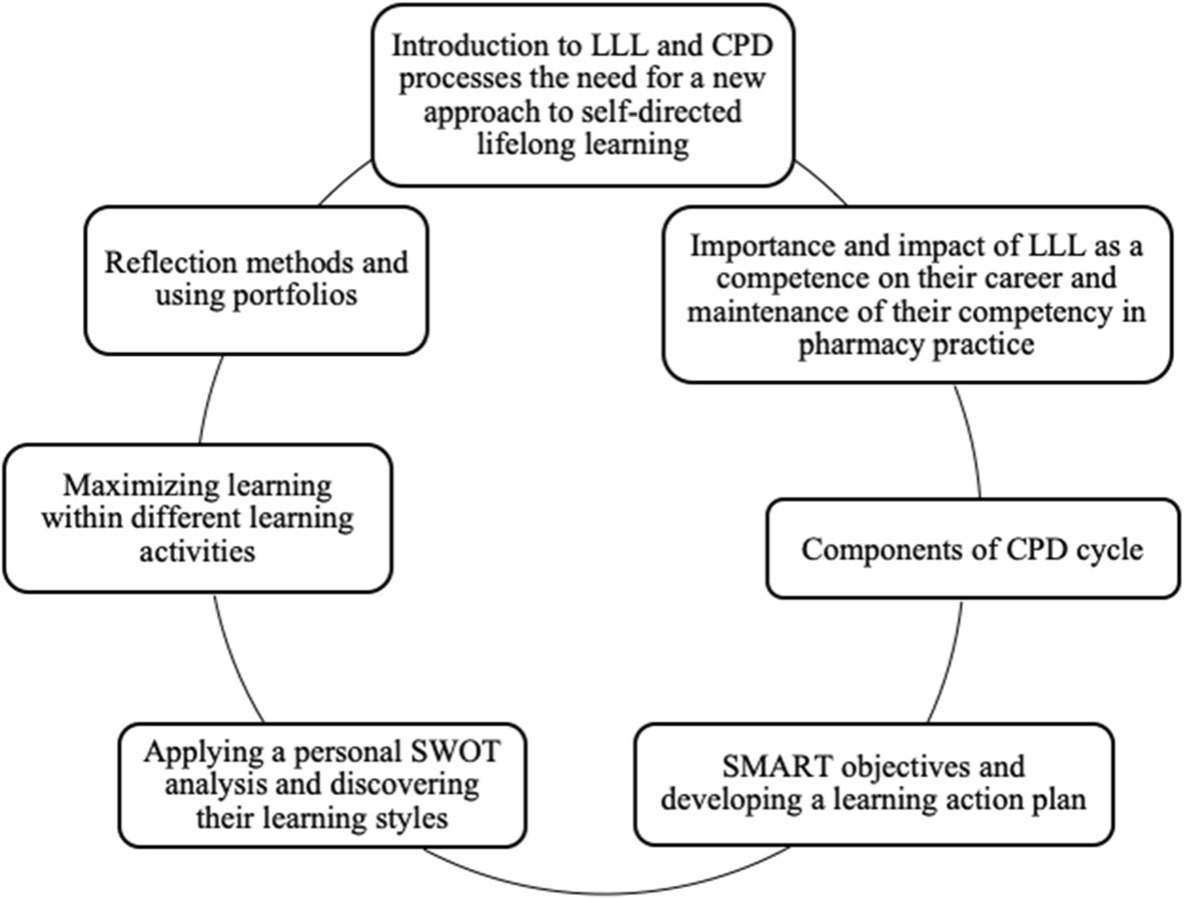
CPD Course content

In the students’ orientation course, the course was delivered as interactive didactic lectures and workshops. The students were provided a 2-h lecture with training on a weekly basis (see Fig. 2).

In the second part of the CPD course, students were requested to determine and address their learning needs based on individual self-assessment during their final experiential internship. Variety of activities and resources including online courses, regional conferences, seminars, workshops, learning materials as videos, textbooks, brochures etc. were all accepted as activities that may help achieve one’s own learning targets. A virtual meeting was held to answer student’s questions and provide guidance to students regarding how to fulfil the required assignments. The students were also provided written guidance on the course description and the answers for expected questions.

### Assessment and evaluation

#### Weekly activities and assignments

During the orientation course of the first semester, weekly assignment activities were required from the students individually or in groups as a formative assessment to achieve course objectives. Following each assignment or homework task, instructors discussed the assignments with students in class to elaborate on their performance and reinforce positive responses. Weekly assignments had scores that represented 5–10% of the total mark of the course.

#### Student’s portfolio

**Fig. 3:**
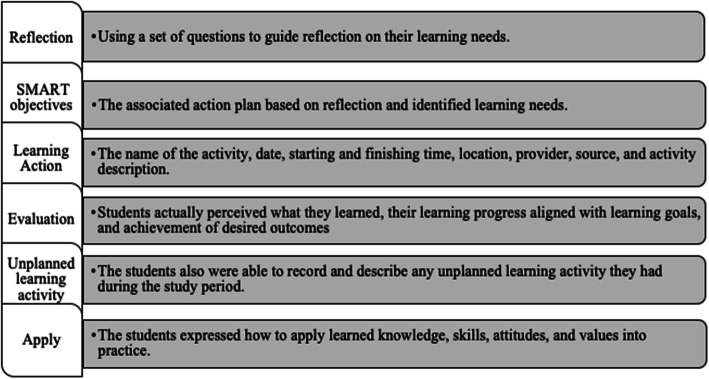
Portfolio sections

Students were required to complete 3 CPD cycles throughout the year: the first cycle was in the first semester, and a minimum of two cycles per student were required in the second semester. For each cycle, each student was required to use a minimum of two different learning activities which were then documented in student’s e-portfolio (see Fig. 3). A validated rubric was used to evaluate the portfolios by the research team. The rubric involved the following items (reflection, SMART objective plan, learning activity, evaluation, application). Each CPD cycle assignment in the second semester formed 20% of the total percentage of the course (total of 40%).

#### Students’ preparedness for LLL (pre-post self-assessment questionnaire)

A comprehensive literature review was conducted to develop the CPD course content and an assessment tool. The developed questionnaire tool consisted of 5 sections and 59 questions recorded on a five-point Likert-type scale. The tool was developed and validated using the Delphi method followed by pilot testing and exploratory factor analysis using a sample of 521 students in the last year of pharmacy programs from 7 countries. The self-administered questionnaire tool was used to assess changes in students’ self-evaluation of their preparedness for CPD and LLL. The questionnaire involved awareness associated with CPD and LLL, SDL skills and attitudes, the practice of CPD cycle components and activities in the past months, motivation factors and perceived barriers to participation in CPD activities.

#### Students’ evaluation of the CPD course (focus groups)

Qualitative feedback was obtained from students using the focus group (FG) approach at the end of the study (see Table 1). The focus group sessions were done in the same format to allow for potential comparisons between groups during the analysis. The facilitators of the sessions were trained in acquiring responses and handling qualitative sessions. An independent observer that took detailed notes and observed the group dynamics was present during the sessions. Each focus group lasted from 30 to 40 min and all sessions were tape-recorded and subsequently transcribed verbatim by an independent experienced transcriber and translator.

**Table 1:** Qualitative Feedback from Student Evaluation of CPD Course (Focus Group Session)

Questioning route	A semi-structured questioning route was developed by the authors and used for three developed groups.
Session questions	1. The course settings (aim, achievement, content, organization, time, assessment methods and instructors), 2. Their experience of skills development (e.g. SMART objectives plan, personal SWOT analysis, learning styles, Curriculum Vitae (CV) development and personal portfolios), 3. Benefits and strengths of the course in enhancing student learning, 4. Barriers and weakness of the course that hindered students’ learning, 5. Experiences students enjoyed most in the course and their suggestions for improving the courses in the future.
Student focus groups (FGs)	Three homogeneous student FGs were arranged based on the preferred medium of communication; a. FG1 and FG2 were conducted in Turkish language b. FG3 in English language.
Informed consent	a. Before the commencement of the focus group, students were asked if they would be willing to participate in an approximately 30-min session to provide feedback on the CPD course. b. All participants were informed that their session will be recorded and assured that their lack of participation in the session would have no effect on their grade.
Qualitative data manipulation	The first stage involved transcription carried by the principal researcher and reviewed by 2nd author for accuracy and annotated for nonverbal content.
	Following transcription, the script was translated into English using backward and forward translation method done by the principal researcher and the 2nd author (bilingual English, Turkish); then by a professional translator (bilingual with Turkish as a first language)
	Following translation, the third stage involved content analysis of the data sets to develop categories and themes.
Inductive thematic analysis	Inductive thematic analysis of the transcripts was undertaken based on six steps [28]^:^ a, becoming familiar with the data; b, generating initial codes; c, searching for themes; d, reviewing themes; e, defining and naming themes f, finally producing the report. - The principal researcher reviewed all the transcripts several times, coded the data and extracted the main emerging themes. - A second investigator reviewed the transcripts and the key themes thus strengthening the validation of study results. - All authors discussed the themes, codes, similarities, and differences until agreement was reached on the key themes and subthemes.

### Data analysis

The statistical analysis of the quantitative data was conducted using SPSS version 23.0 (IBM Corporation, US). Categorical variables, such as gender, age, nationality, and future plans, were presented in frequencies and percentages. In addition, the continuous variables of the pretest and posttest scores of the CPD simulated program course were expressed as the mean ± SD, and the unpaired t-test was used to compare the control and the intervention groups. The paired t-test was applied to determine the mean and median significant differences between the pretest and posttest scores of the intervention group. *P*-value < 0.05 was considered statistically significant.

Thematic analysis was employed to inductively and deductively derive themes from qualitative data using the NVivo 12.6 software (QSR Intl Pty Ltd.; Doncaster, Australia).

## Results

### Students and participants’ characteristics

103 fifth-year students were invited to complete a cross-sectional self-administered questionnaire, of which 67 (65%) responded. 40 (59.7%) students from among the respondents were randomly selected and invited to join the course, of which 27 (67.5%) students registered and completed the course while the other 13 (32.5%) were not able to register for the course. Of those 13, seven (53.8%) of them were transfer students who still had extra lessons to complete from the previous years, four (30.8%) of the students were international students who could not attend conferences and other activities in Cyprus and Turkey due to the travel and language barriers, and two (15.4%) were in their graduation semester.

Meanwhile, the remaining 40 (59.7%) students were invited to fill the SPLLL questionnaire at the beginning and the end of the academic year. Only 27 students responded to the questionnaire at the end of the study. The cumulative grade point average (cGPA) of students in the study group was 2.35 ± 0.39; which showed no significant differences compared to the mean cGPA of the class (2.35 ± 0.39 vs 2.45 ± 0.36, *p* > 0.05). The characteristic data of the intervention group students are summarized in Table 2.

**Table 2:** Students’ Demographic Data (*N* = 27)

Variable	(%)
Gender	
Male (*n* = 10)	37
Female (*n* = 17)	63
Age	
20–25 (*n* = 26)	96
26–30 (n = 1)	3.7
> 30 (*n* = 0)	0
Nationality	
Turkish (*n* = 19)	70
Cypriot (*n* = 4)	14.8
Nigerian (n = 1)	3.7
Iraqi (n = 3)	11
Future Plan	
Community Pharmacist (*n* = 22)	18.5
Hospital Pharmacist (n = 3)	11.1
Clinical Pharmacist (n = 4)	14.8
Industrial Pharmacist (n = 3)	11.1
Academic (Master, Ph.D.) (n = 5)	18.5
Marketing (n = 1)	3.7
CGPA	
3.5–4 (*n* = 1)	3.7
3–3.5 (n = 1)	3.7
2.5–3 (*n* = 5)	18.5
2–2.5 (*n* = 16)	59.3
1.5–2 (n = 4)	14.8
PILS^a^	
Assimilator (*n* = 11)	40,7
Diverger (n = 9)	33.3
Accommodator (n = 4)	14.8
Converger (n = 3)	11
Having CV (n = 17)	63

^a^Pharmacist’s Inventory of Learning Styles (PILS)

### Students’ assignments and portfolios

Out of the 27 students enrolled in the course, 8 (29.6%) students completed all the weekly assignments. Regarding the portfolio, 18 (66.7%) students submitted two fully completed CPD e-portfolios, and the other 9 (33.3%) students presented uncompleted portfolios. Table 3 shows the evaluation of the students in the course.

**Table 3:** Students Evaluation on Assignments, Portfolios and Total Grade (N = 27)

	Weekly assignments		Portfolio		Total grade in the course	
	N	(%)	N	(%)	N	(%)
Grade System in NEU						
3.5–4	15	55.6	13	48.1	12	44.4
3–3.5	8	29.6	6	22.2	9	33.3
2.5–3	4	14.8	3	11.1	3	11.1
2–2.5	0	0	2	7.4	2	7.4
1.5–2	0	0	0	0	1	3.7
1–1.5	–	–	3	11.1	–	–
0–1	–	–	–	–	–	–

### Students’ preparedness for LLL scale (pre-post self-assessment questionnaire)

No significant differences were found between the study group and other fifth year students in the students’ self-assessment using the SPLLL scale compared to the baseline, whether in total score (166.2 ± 15.2 vs 161.62 ± 16.72; *p* > 0.26) or the domains of the scale, except in the attitude scores that were higher in the study group. Following the implementation of the course, students’ assessment scores were significantly higher overall and for all scale domains compared to the baseline assessment, as shown in Table 4. Additionally, compared to fifth year students who responded to the second SPLLL questionnaire at the end of the study, students who enrolled to the course were rated significantly higher in knowledge, skills, and practice associated with LLL compared to the control post intervention (*p* < 0.02).

**Table 4:** Pre and post subscales for intervention group (N = 27)

	Range	Pre-test score	Post-test score	Change in score (%) M (SD)	*P* value
		M (SD)	M (SD)		
Subscales					
Knowledge	14–70	40.85 (6.55)	60.8 (8.89)	29 (16)	.000
SD skills	12–60	44.2 (6.53)	51 (6.04)	11 (13)	.000
Attitude	13–65	49.44 (6)	54.4 (6.7)	8 (13)	.000
Practice	12–60	31.67 (4.87)	46.56 (8.69)	25 (14)	.000
Total	51–255	166.2 (15.2)	212.78 (27.1)	18 (11)	.000

### Students’ evaluation of the CPD course (focus groups)

Three FGs were formed. 21 out of 27 students participated (FG1 *n* = 9, FG2 *n* = 8, and FG3 *n* = 3) in the focus group sessions conducted at the end of the academic year while 6 students declined to participate due to scheduling conflicts or other work priorities.

Four themes emerged from the latent content analysis: 1) the course framework and factors influencing the course effectiveness, 2) SDL and professional development skills, 3) the portfolio and 4) recommendations. Following the transcription and coding of all focus group sessions, out of the codes identified, four themes emerged. Each of these themes and their codes is presented with participant quotations included to illustrate them (Table 5).

**Table 5:** Students’ Evaluation of CPD Course (Focus Group Sessions)

Objectives and themes	Codes	Feedback	Related Statements
*The course framework*	Aim of the course	Students in each group were asked if they agree that the course contents match with the aim of the course “to improve and develop pharmacy students’ CE and professional skills to become lifelong learners”. All groups agreed that the aim and objectives of the course match the course content with an overall rating of 85%.	*“This course was beneficial, at the beginning we learned how to assess ourselves and how to determine our weakness and strength, then how to select the appropriate seminars and other necessary activities to improve ourselves”. FG1* *“At the beginning, I was worried because I heard that we need to attend seminars and it’s hard for me as I am not from that type of person. But later on, I attended and it became beneficial”. FG1*
	Course objectives achievement	In terms of the course objectives achievement, the extent of achievement out of 100 varied among groups. FG1 and FG2 gave 95 and 85% respectively, the international students’ FG3 rated 65% of course objectives to be achieved. According to some students, the bilingual nature of lectures was a barrier to achieving the course objectives as it caused them to lose focus. The second main barrier was the lack of student’s time especially transfer students who had extra lessons from previous years thus less time to do assignments.	*“Bilingual lectures are hard to follow”; “we didn’t have time”. Although many other students represent the achieving of the aim as “I got benefit and I know how to improve myself now”. FG3*
	Course organization	The overall rating was 85%. There are many sub-codes under the course organization based on the groups’ responses. a. Regarding the timing of the orientation lessons, student’s views varied, yet the majority of the students preferred the early morning time for lectures and workshops. b. The second sub-code identified was the sufficiency of information provided about the course before students’ registration. According to FG1 feedback, one of the major limitations in the course organization was insufficient information being provided about the course prior to their registration	*“lesson time and organization were good”. FG3* *“The other mornings’ lessons are not interactive, but this lesson needed interaction which was hard in early morning”. FG1* *“We heard you need only to attend 2 conferences and you will finish. But later on, we took lectures every week Friday 09:00 am”. FG1*
	Course delivery method “Individual-based learning needs”	The course delivery method was positively rated by the students in all groups. The students liked the interactive teaching method adopted as well as the workshops and in-class discussion led by the instructors. Students perceived the course delivery method as an “effective way to learn, share, apply and develop a skill”. They were satisfied with the material content and references as well and they embraced the need for more interactive and group work learning in pharmacy education curriculum. Students also pleased that the course was individual-based and addressed their own learning needs.	*“at the beginning, there was theoretic lecture and explanation then we applied what we learned, it was good”. FG1* *“There were many in-class activities, also slide presentation/material were attractive. The group and the friendly environment work were great; it was a good and beneficial course”. FG1* *“Teaching with group work in the pharmacy, help in achieving your aims and everything. Now I am planning to open a community pharmacy, and I know how to develop myself. It was a realistic course, and it showed us that everyone learned something different than others”. FG1* “*I felt myself a master student. I got used to sleeping in many lessons, but in this course, I did not*”. *FG1* *“Everyone assessed his weakness and need individually, then accordingly we improved, it was like private lesson”. FG2*
	Course assessment and assignments activities	Students rated the assignments as to achieve 90% of their educational objectives. The topics to practice weekly assignments or activities were selected by the students based on their educational need; this helped them to fill previous gaps in their learning. Students were highly pleased with the in-class discussion of homework and assignments, as well that the course assessment wasn’t based on exams which motivated their learning more than courses with exams that they see stressful and not properly represent their actual learning. In FG3, students stated barriers that hinder them from doing assignments; these included the lack of enough time for carrying all self-directed assignments. Also, students in FG3 found it hard to determine activities to attend such as conferences, seminars, and workshops as activities are rare within university and in North Cyprus. Also, the registration fee for those available activities was a barrier for them as students to attend.	*“we liked assignments, we selected the topics that we want, searched and then discussed it in groups. It was beneficial”. FG2* *“in other courses, we are doing our homework and waiting for the grade, but in CPD we discussed with both instructor and students”. FG2* *“in the conferences and seminars, we were there to learn, we are not worried about the exam or what they will give in the exam”. FG1* *“yes, it was hard to do the activities and fill it because we don’t have time”. FG2*
	Course instructors	The overall evaluation of FG1, FG2, and FG3 for the instructors was 100, 100, and 90% respectively. Students evaluated the instructor to be a good communicator, used eye contact, helpful and understandable. The groups agreed that the instructor was professional, knowledgeable, and well prepared, which facilitated achievement of course objectives.	*“we were able to contact him from anywhere in anytime and he was answering our queries”. FG1* *“instructors inspired us when they shared with us their real stories, their aim and how to do a plan and how to change or improve ourselves. When I’m thinking, all these things I have gotten are from the course”. FG1*
	Whether they recommend this course in pharmacy education curricula or not	The students were asked whether they recommend this course in pharmacy education curricula or not, all answered by *“yes, we strongly recommend 100%”.* Students were also asked about their thoughts regarding the most appropriate semesters to start CPD course. Different opinions were brought out and a discussion took place between the students for a while. Even though all students reached a deal that this course is necessary for students before graduation, few students agreed that course should be delivered the last year proceeding graduation. Some students expressed their belief that this course in its current format is challenging for the fifth year students during their final internship course as they are also writing graduation thesis. The big discussion was about the effectiveness of having this course in early years not only the last year, most students supported the idea that CPD should be taught earlier in curriculum.	*“yes, strongly recommended 100%”. FG3* *“we think 5th is most suitable to assess and improve ourselves before graduation after almost finishing all courses”. FG1* *“it was good for the 5th year students in the 1st semester, but it was not good for them in the 2nd semester in term of time”. FG2* *“we wished it was on other years, 4th or 3rd year maybe we would do better and it’s more logic, but not at the last year”. FG2* *“1st or 2nd year because when they started to attend conferences they are going to a trip not to learn, so I think it’s good for them to learn from the beginning, there was a lot of free time in these years”. FG1*
	Duration of the course	FG3 agreed that two semesters are enough for such a course, while students of FG1 and FG2 recommended that this course should be delivered continually starting from the early years until graduation. Some students stressed on the importance of having it from the early years.	*“CV should be prepared from 4th year, but conferences and activities should be before. 4th year is late, in our opinion from the 3rd year”. FG2*
	Elective or compulsory course;	Students when asked about the status of this course in curricula whether it keeps as an elective or become a compulsory course, all students recommended to deliver the course as a compulsory course for many reasons they stated.	*“something that everybody should know, so should not be an elective course but compulsory”. FG3*
Acquired SDL learning and professional development skills		During the session students reflected what they had gained from this course and the differences they noticed on their learning on individual bases. Students were pleased that they have their curriculum vitae (CV) and they can develop it by themselves. Students were also pleased that they practiced how to assess and address their learning needs and using online learning resources effectively.	*“before I was attending activities only for attending, but now first I need to find what I need then I will attend after having my plan. It was an opportunity for us to learn it”.* FG2 *“the CV, we didn’t know well before, but now everyone had his CV”.* FG2 *“because most of my friends were asking me to teach them how to make their CV, I was proud and I was like okay I knew how to do it in class and I’m going to teach you”.* FG3 *“I was able to learn what I’m weak in from the internet but before I did not use to”.* FG2
Portfolio		Students were asked about their thoughts about the portfolio they used and whether it was beneficial. FG1 rated portfolios 85% in terms of utility and content, while FG2 and FG3 evaluated portfolio to achieve only 55% in terms of easiness to use and applicability*,* although they found that using portfolios is beneficial. Regarding the format of the portfolio, most of the students liked the e-portfolio however, some of the students preferred the hard copy format perceiving it to be more beneficial than the online version.	*“we felt boring a lot of repetition in the questions, some questions sound as being repeated and lots of details. It’s better to be briefer”.* FG2
Recommendations		At the end of the focused group sessions, we asked the students about their recommendations to improve the course. a. The first recommendation was about the time of the lesson within the day, not to be very early. Also, students recommended starting CPD course earlier in curricula. b. The second recommendation was about announcement, suggesting course directors to provide them information of potential learning activities, conferences, seminars or any learning activities offered in nearby places. c. The third recommendation was to deliver the course in one language instead of being delivered bilingual using both English and Turkish languages. d. The fourth recommendation was related to the portfolio; students recommended shortening the portfolio and making it briefer. e. Other suggestions involved cooperating with other departments to provide more learning activities or opportunities including interprofessional activities (e.g. with the medicine faculty) within university campus with proper prior announcement. Students suggested finally to develop a faculty calendar that shows all learning activities in the region and within school.	*“better time fitting our schedule”.* FG1 *“we need to know this information before the last year”.* FG2 *“I really felt bad, even I couldn’t communicate with my friends”.* FG3 *“we think pharmacy faculty should host many activities as conferences”.* FG1 *“I would add more activity inside the class, and announce more conferences for the students to attend”.* FG3 *“a calendar of the planned conferences in Turkey and Cyprus would be helpful”.* FG2 *“the first semester was good but the second one was hard especially for the students training in Cyprus”.* FG1

## Discussion

The implementation of the CPD simulation course resulted in higher mean scores on the SPLLL scale compared to their classmates and their self-rating before implementation. The course provided students with opportunities to practice and develop skills in self-assessment and awareness, SMART planning, evaluation and proper documentation of their learning, which are all desirable for LLL. Most students performed very well (78%) in their assignments and got high scores on their portfolio evaluation. Students perceived that the course matched its aim and that they had achieved most of the course objectives. Students perceived themselves currently more committed and oriented to LLL and professionalism.

CPD and LLL in pharmacy education is challenging, inconsistent, and usually not assessed or even required in many pharmacy programs in Cyprus, Turkey and across the globe. In this study, grounded theoretical features were employed within a longitudinal CPD course to enable students to develop themselves as independent lifelong learners beyond graduation.

Within the different learning modes, educators identified varying advantages and disadvantages associated with each mode of learning. In the current course, a wide range of teaching methods were adopted involving exposition, discussion, enquiry, activity and collaboration [29].

A small group learning method was used to enable enhanced knowledge exchange and discussion among students and with their instructors. Small group learning is well established in the literature as an effective setting for learning [29] and a method preferred by pharmacy students suitable for enhancing LLL skills [29, 30].

The CPD cycle derived from Kolb’s learning cycle [17] was adopted as a main framework for students’ assignments and portfolios. In the literature, students’ completion of a minimum of two CPD cycles was reported as an effective utilization of the mode in leadership and professional development [31]. In the current study, a minimum of three completed CPD cycles was required to assure students’ competence in utilizing the CPD cycle.

Other features possibly contributing to program outcomes include a lengthy course duration in contrast to short courses or workshops shown by many educators to be less effective and having an effect that may last a week or a few hours [32, 33]. Active learning methods known to improve the problem-solving and critical thinking skills of students along reflective portfolios that provide evidence of professional development and the achievement of the desired competencies were all adopted during the course [12, 34, 35]. Both subjective and objective assessment methods were utilized. Based on Donald Kirkpatrick’s developed model to evaluate the overall effectiveness of training programs [21].

Several students stated that they found the SMART learning objective exercise to be useful and beneficial. One of the students stated that “*I guess I learned especially from the SMART objective and personal SWOT analysis, I even used it in my scientific presentation course. I was able to talk about how to be specific and to be smart in planning everything that you do in pharmacy, achieving your aims and everything*”. This perception toward the SMART learning objective was similarly reported in US schools [14].

CV development is an important ability closely linked to CPD, as also emphasized by Dyke JE et al.’s study. Students were suggested to design and update their CVs during the course so to grasp how CPD may contribute toward improving their CVs fast with time [18].

Students found portfolio development to be one of the more challenging activities in the course. A student said that “*it was stressful and needed concentration, but it’s beneficial*”. A similar study reported that 40% of the surveyed students found the portfolio to be a challenge while 54% of them reported that it was effective in supporting their learning [36]. The use of online training modules and electronic portfolio submissions made the CPD program much more convenient. A student commented that “*using online portfolio and incorporating technology was pleasant and unexpected*”.

CPD is not learning for the sake of learning; it helps to move students toward their career goals [9]. As a student expressed in the focus group session, that now they can improve themselves in all fields: “*now we also might improve ourselves not only in a community pharmacy, we could work also in other sectors*”. CPD allows students to individualize aspects of their education [18] since being a self-directed lifelong learner requires skills for determining individual learning needs. The students reflected on how they liked that the course was based on individual learning needs: “*in university things are not based on our weakness or need we are never asked this. But in CPD the activities were based on our needs and then we improved that was good*” as a student in FG1 emphasized.

O’Brocta et al. suggested that incorporating the CPD process early in the 1st year will familiarize the students with the CPD method and permit them to become more proficient in applying it. Continuing the CPD process during advanced experiential years mimics its integration into actual pharmacy practice [37]. Students in the current study supported these opinions, where most of them preferred to have orientation in the early years while the practice of CPD should be required in the advanced years of the program: “*there are basic information we could have even from first classes, such as why CPD, why we need it, and some online courses, while maybe some advance things as portfolios and conferences are suitable for 5th-year students, but at least basics can be delivered earlier*” as a student commented.

Improving the knowledge of students’ learning preferences, behaviors, and strategies can benefit and guide CPD. Applying Austin’s Pharmacist’s Inventory of Learning Styles tool can contribute to defining, describing, and measuring learning styles among pharmacists [38]. The dominant learning style of the students in current study was assimilator (40.7%), followed by diverger (33.3%), accommodator (14.8%) and converger (11%). A similar distribution was reported in a study done at the University of Malaysia involving pharmacy students in which the dominant learning style of the students was assimilator [39].

A few limitations of the current study are mentioned. To start with, the small sample size for the students limits the generalization of the study findings over the study population. Additionally, the response rate of the 5th year students used as a control was not high enough, although the current responses are considered acceptable for generating hypotheses [40]. Further, the subjective nature of the self-evaluation, as in the case of SPLLL scale used in this study, may be considered as a limitation, although an objective assessment of assignments and portfolios by instructors was done. Additionally, it is important to mention that pre-post assessments could be subject to recall bias, though the duration between the two assessments in the current study was relatively long (9 months). Also the multifaceted nature of CPD processes utilized in different countries may arise challenges in replicating this course, yet the process adopted in this course is universal and promoted by the world FIP [10].

Despite the presence of these limitations, the findings of this study contribute to the prior literature on LLL and CPD in pharmacy education. To date, no researcher reported the successful implementation of an LLL targeted program utilizing the CPD model in programs outside the US. Dyke JE et al. reported poor outcomes of the program at a UK based university. In addition, in an Australian university, although an improvement of students’ skills was noticed, poor student acceptance of the TLR was reported [18, 19] contrary to the current study findings. The introduction of the CPD simulation in an advance year coupled with experiential practices is contrary to Dyke JE et al’s course, which was administered to first year students, and this may explain the success of the program in North Cyprus. Additionally, the small group learning strategy adopted and the lower complexity of the program introduced in the current study may justify higher student satisfaction and acceptance compared to the TLR study. Other features supporting the validity of the findings of the current study include the mixed method design adopted to generate both quantitative and qualitative data. In regard to the course assessment, both objective and subjective approaches were used to evaluate student performance and all components of the program. The course features were mainly supported by grounded theories and evidence. This study is the first to report an attempt to implement longitudinal courses targeting and developing CPD and LLL in resource-limited settings or developing countries.

Future research must assess the implementation and impact of similar programs using a larger sample of students, especially for the early introduction of the program in the second and third years of M.pharm programs coupled with introductory pharmacy practice experiences. Practicing CPD within experiential courses is important since it simulates the required setting of pharmacy practice as the students graduate. Assessing the impact of similar programs following student’s graduation and registration as practitioners would be useful too.

The implementation of a CPD course may also provide more flexible opportunities or a window for learning newly evolving concepts or practices not addressed in pharmacy curriculum since curricula needs years to be revised and updated in many countries. Students in the current study have reported that their self-development in both areas were not sufficiently addressed during their studies and in new areas previously unfamiliar to them (e.g., sports medicine and vaccinations). Thus, assessing such an impact of a CPD course in contrast to other courses provided within curricula may further enrich the current literature.

## Conclusion

The implementation of a CPD simulation course improved students’ knowledge, skills, attitudes and practice of CPD, evaluated using a self-assessment scale (SPLLL). The course provided students with opportunities to practice and develop skills which are desirable for LLL. Students well perceived the setting of the course and recommend to introduce the course earlier as a mandatory course in their curriculum. Future work should focus on the early introduction of similar programs and its impact on future pharmacists’ post registration and in practice.

## Data Availability

The data sets supporting the conclusions of this article are available in excel file and can be provided if requested.

## Abbreviations

CPD: Continuing professional development
LLL: Lifelong learning
SPLLL: Students’ preparedness for lifelong learning
FIP: International Pharmaceutical Federation
PharmD: Doctor of pharmacy
US: United State
ACPE: Accreditation Council for Pharmacy Education
RUCOP: Roseman University College of Pharmacy
TLR: Traffic light report
M.pharm: Master of pharmacy
CE: Continuing education
NEU: East University
SDL: Self-directed learning
FG: Focus group
CV: Curriculum Vitae
CGPA: Cumulative grade point average
PILS: Pharmacist’s Inventory of Learning Styles
